# Minimally invasive treatment of cirrhotic secondary hypersplenism with high-intensity focused ultrasound

**DOI:** 10.1038/s41598-022-24416-x

**Published:** 2022-11-30

**Authors:** Xiumei Zhang, Shilin Tang, Guohua Huang, Zhihong Xu, Caiju Feng, Gaowu Yan, Suyu He

**Affiliations:** 1 Zunyi Medical University, Zunyi, China; 2 The High Intensity Focused Ultrasound Center, Suining Central Hospital, Suining, Sichuan China; 3 The Department of Radiology, Suining Central Hospital, Suining, Sichuan China; 4 The Fourth Department of Digestive Disease Center, Suining Central Hospital, Suining, Sichuan China

**Keywords:** Portal hypertension, Outcomes research, Liver diseases

## Abstract

High-intensity focused ultrasound (HIFU) has been reported to be a minimally invasive effective method for the treatment of secondary hypersplenism. However, neither the short-term efficacy nor the indications and/or contraindications have been described in patients with cirrhosis. From October 2019 to May 2021, eleven cases of patients with cirrhotic secondary hypersplenism were enrolled. The blood counts, liver function tests and abdominal ultrasound and/or MRI scans of all patients were closely evaluated. Among these 11 patients, eight (72.7%) patients were classified as Child–Pugh A, and the other 3 (27.3%) patients were Child–Pugh B; Five (45%) patients were diagnosed with gallstone, including multiple small stones in 2 patients and single stone in 3 patients. HIFU was performed successfully in all 11 patients. After HIFU, hematologic parameters and liver function were significantly improved in all 11 patients (*p* < 0.05). The HIFU ablated volume to spleen volume rate was 35–61%. Complications were ecchymosis of the waist in 7 (63.3%) patients, ablated area pain in 3 (27.3%) patients, and choledocholithiasis in 2 (18.2%) patients with multiple small gallstones. All of them recovered smoothly without additional treatment except for 2 patients with choledocholithiasis recovered with risky endoscopic retrograde cholangiopancreatography (ERCP) treatment. This series suggested that HIFU is an effective and safe treatment for cirrhotic secondary hypersplenism in patients classified as Child–Pugh A or B. However, multiple small gallstones could be a relative contraindication for it.

## Introduction

In clinical practice, second hypersplenism caused by cirrhosis is very common. From invasive methods (splenectomy) to minimally invasive methods (partial splenic embolization/radiofrequency ablation), doctors and investigators try their best to address cirrhotic hypersplenism with the least harm to patients with the hope of obtaining the most benefits^1–3^. However, since secondary hypersplenism often occurs in patients with decompensated cirrhosis, the patients’ poor condition combined with hyperkinesis of the portal vein and fragile spleen tissues make not only the recovery of the patient long and difficult but also the incidence of complications high. Thus, many investigators have tried to explore an alternative and noninvasive method for the treatment of second hypersplenism caused by cirrhosis.

High-intensity focused ultrasound (HIFU) was introduced in the 1940s as an approach for thermal ablation of viable tissues^4^. As an emerging noninvasive therapy, HIFU has been successfully applied to treat solid, well-defined tumors, including those in the pancreas, liver, uterus, and prostate^4–6^. In recent years, HIFU has also been applied and proven to be safe and effective in the treatment of second hypersplenism caused by cirrhosis^7^. Although previous studies proved the safety and efficacy of HIFU in the treatment of secondary hypersplenism, neither the appropriate indications nor the relative contraindications for HIFU in the treatment of hypersplenism caused by cirrhosis have been clearly pointed out. To identify appropriate patients with hypersplenism who may receive the most benefit at the least cost from HIFU treatment, this study evaluated the complications and short-term efficacy of cirrhotic patients with secondary hypersplenism who underwent HIFU treatment at our single center.

## Results

Eleven patients with a diagnosis of hypersplenism secondary to cirrhosis were treated with HIFU. Their average age was 54.0 ± 8.62 years, and males comprised the majority (Table 1). The cause of cirrhosis was chronic hepatitis B infection in 9 patients and alcoholic hepatitis in 2 patients.

**Table 1:** Patients’ demographics, clinical presentation, abdominal ultrasound and gastroesophageal endoscopy.

Parameters	n (%)
**Patients’ characteristics**	
Age (years), mean ± SD	54.0 ± 8.62
Male	8 (72.7%)
**Cause of cirrhosis**	
Hepatitis B	9 (81.8%)
Alcoholic hepatitis	2 (18.2%)
**Clinical presentation**	
Ascites	2 (18.2%)
Hematemesis	6 (54.5%)
Left upper abdominal pain	3 (27.3%)
Epistaxis	3 (27.3%)
Ecchymosis	4 (36.4%)
**Child–Pugh grading**	
Child–Pugh A	8 (72.7%)
Child–Pugh B	3 (27.3%)
**Ultrasound**	
Splenomegaly	11 (100.0%)
Gallstone	5 (45.5%)
**Gastroesophageal endoscopy**	
Esophageal varices	11 (100.0%)
Grade I/II/III	2/5/4

### Pre-HIFU

All patients had portal hypertension, leukopenia and thrombocytopenia. The mean WBC count was 3.1 ± 0.61 × 10^9^/L, and the mean PLT count was 26.4 ± 5.64 × 10^9^/L. According to the Child–Pugh classification, the liver function of 8 of 11 patients was classified as Child–Pugh A, and that of 3 patients was classified as Child–Pugh B. Abdominal ultrasound and/or MRI scans showed cholecystolithiasis in five patients. Two patients had chronic cholecystitis with multiple small gallstones, and the other patients had a single large gallstone. Among all 11 patients, 6 had a history of esophageal and gastric variceal bleeding.

### HIFU and complications

**Figure 1:**
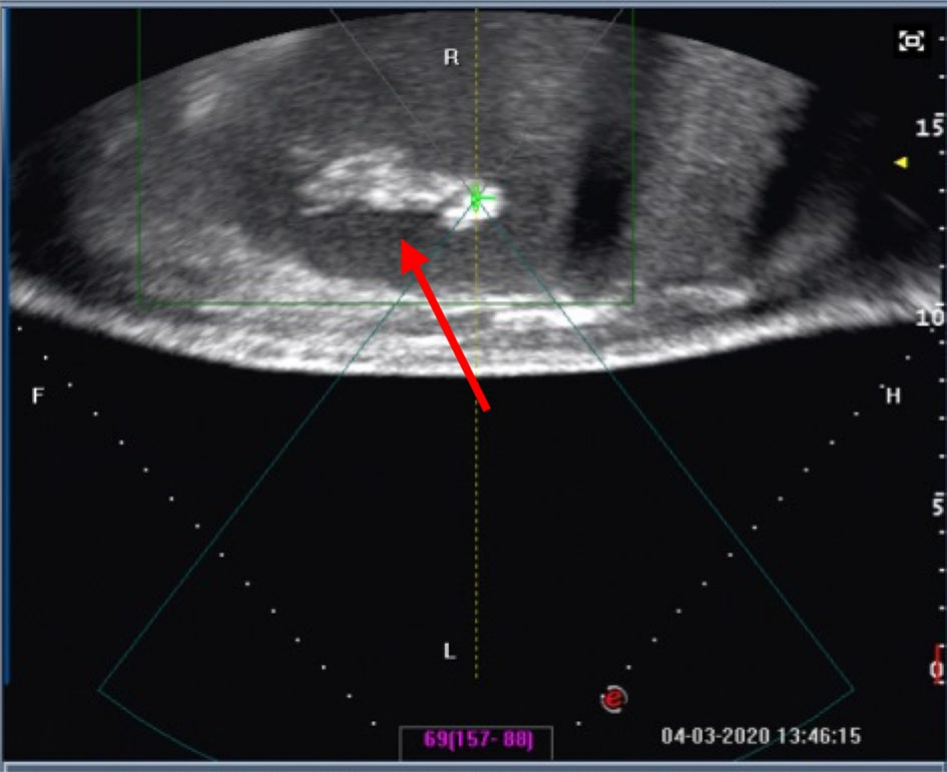
Real-time grayscale sonographic changes during high-intensity focused ultrasound treatment. The real-time sonography showed a hyperechoic area in the HIFU treated region of the spleen (red arrow).

**Figure 2:**
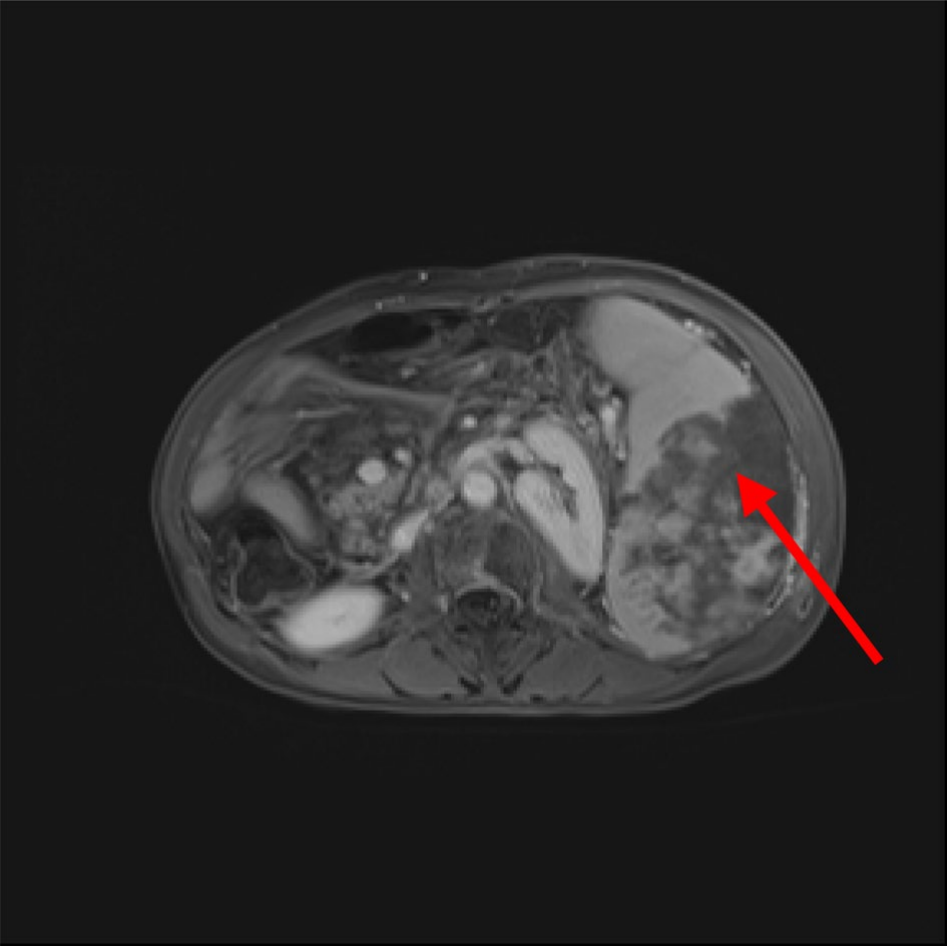
Contrasted MRI showed a patchy nonperfused area of the spleen two weeks after the HIFU treated region of the spleen (red arrow).

**Figure 3:**
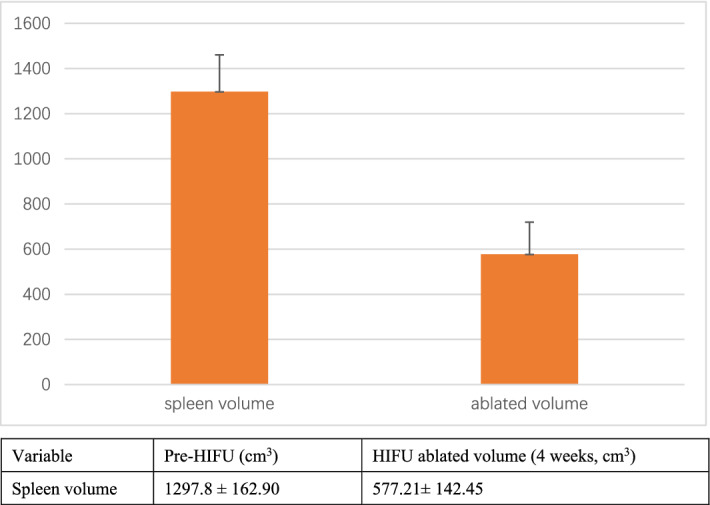
The pre-operative spleen volume and the HIFU ablated volume.

HIFU was successfully performed in all 11 patients. The real-time sonography showed a hyperechoic area in the HIFU treated region of the spleen during the treatment (Fig. 1). The ablated area presented as a patchy nonperfused area of the spleen in contrasted magnetic resonance imaging (MRI) (Fig. 2). The spleen volume was 1297.80 ± 162.90 cm^3^ before HIFU, and the ablated volume was 577.21 ± 142.45 cm^3^ (Fig. 3). The ablated volume to spleen volume rate was 35–61%.

Post-HIFU, three main complications occurred. First, skin lesion. Subcutaneous ecchymosis of the waist was observed in 7 (63.6%) patients. Second, ablated area pain. Three (27.3%) patients complained of mild pain in the left upper abdomen. All patients recovered smoothly with proper symptomatic treatment. Third, choledocholithiasis. Two patients who were diagnosed with chronic cholecystitis with multiple small gallstones complained of severe abdominal pain, fever and jaundice on the third day and eighth day post-HIFU. Subsequent MRI showed multiple small stones incarcerated in the common bile duct. The diagnosis of choledocholithiasis was confirmed. Both patients recovered with subsequently anti-infection treatment and endoscopic retrograde cholangiopancreatography (ERCP).

### Post-HIFU follow-up

**Figure 4:**
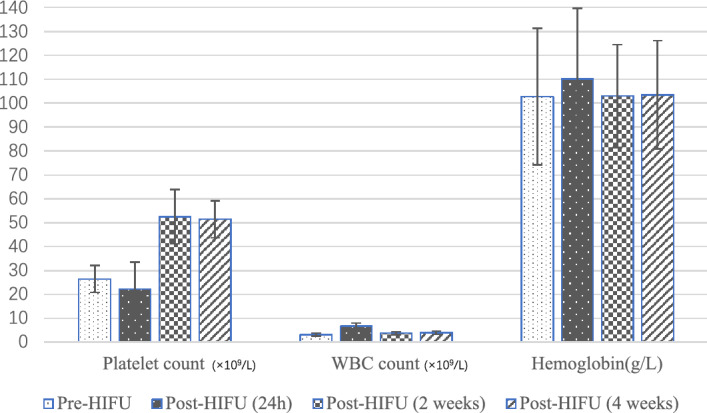
Hematological changes pre-HIFU and post-HIFU.

The trends of average peripheral blood cell count 24 h post-HIFU, 2 weeks post-HIFU and 4 weeks post-HIFU are shown in Fig. 4 and Table 2. The WBC counts tended to markedly increase first and subsequently decrease to a high plateau 4 weeks post-HIFU compared to baseline (*p* < 0.05). Conversely, owing to the postoperative reaction, the PLT counts tended to markedly decrease first and subsequently increase and stay in a relatively high plateau 4 weeks post-HIFU compared to baseline (*p* < 0.001) (Table 2).

**Table 2:** Hematological changes pre-HIFU and post-HIFU.

Variable	Pre-HIFU	Post-HIFU (24 h)	Post-HIFU (2 weeks)	Post-HIFU (4 weeks)
Platelet count (× 10^9^/L)	26.4 ± 5.64	22.3 ± 11.10^#^	52.5 ± 11.42^##^	51.4 ± 7.78^##^
WBC count (× 10^9^/L)	3.1 ± 0.61	6.6 ± 1.37^##^	3.7 ± 0.68	3.9 ± 0.64^#^
Hemoglobin (g/L)	102.7 ± 28.52	110.1 ± 29.61	103.0 ± 21.60	103.5 ± 22.75

*HIFU* high intensity focused ultrasound, *WBC* white blood cell. ^#^Compared with pre-HIFU values, *p* < 0.05.

^##^Compared with pre-HIFU values, *p* < 0.001.

**Figure 5:**
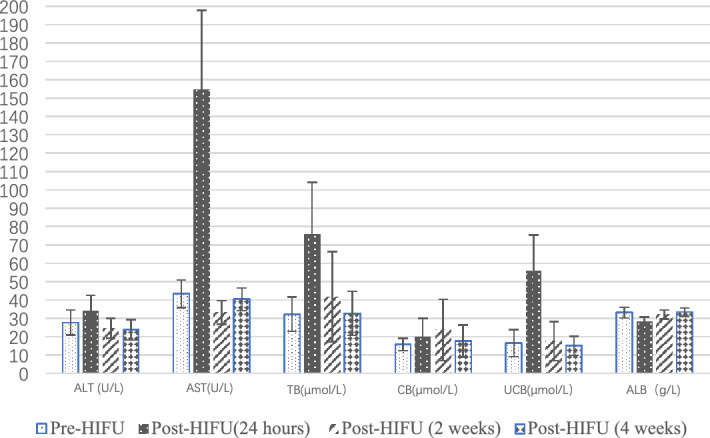
Liver function changes pre-HIFU and post-HIFU.

Changes in alamine aminotransferase (ALT), aspertate aminotransferase (AST), aluminum (ALB) and total bilirubin (TB) post-HIFU are presented in Fig. 5 and Table 3. Compared to baseline, the AST levels markedly increased and peaked 24 h post-HIFU. However, no significant difference in ALT levels was observed (*p* > 0.05). The TB level peaked 24 h post-HIFU (*p* < 0.001) and gradually decreased to baseline 4 weeks post-HIFU. The ALB level tended to decrease sharply 24 h post-HIFU (*p* < 0.05), but gradually recovered 4 weeks post-HIFU. Both conjugated bilirubin (CB) and unconjugated bilirubin (UCB) levels tended to markedly increase first and subsequently gradually decrease to a low plateau. The CB level peaked 2 weeks post-HIFU while the UCB level peaked 24 h post-HIFU. The increase value of UCB was significantly higher than the CB (*p* < 0.001) (Table 3).

**Table 3:** Pre-HIFU and post-HIFU liver function.

Variable	Pre-HIFU	Post-HIFU (24 h)	Post-HIFU (2 weeks)	Post-HIFU (4 weeks)
ALT (U/L)	27.7 ± 6.77	34.1 ± 8.35	24.6 ± 5.46	23.8 ± 5.58
AST (U/L)	43.4 ± 7.57	154.8 ± 42.94^##^	33.2 ± 6.34^#^	40.4 ± 6.24
TB (μmol/L)	32.2 ± 9.37	75.8 ± 28.22^##^	41.8 ± 24.60	32.7 ± 11.98
CB (μmol/L)	15.7 ± 3.32	19.9 ± 10.22	23.7 ± 16.68^#^	17.6 ± 8.81
UCB (μmol/L)	16.5 ± 7.34	55.9 ± 19.42^##^	17.6 ± 10.60	15.1 ± 5.16
ALB (g/L)	33.1 ± 2.92	28.4 ± 2.44^#^	32.1 ± 2.44	33.4 ± 2.17

*HIFU* high intensity focused ultrasound, *ALT* alamine aminotransferase, *AST* aspertate aminotransferase, *TB* total bilirubin; CB, conjugated bilirubin, *UCB* unconjugated bilirubin, *ALB* aluminum.

^#^Compared with pre-HIFU values, *p* < 0.05.

^##^Compared with pre-HIFU values, *p* < 0.001.

## Discussion

Due to its minimally invasive nature, HIFU has attracted much attention in recent years. It has been a mature approach in the treatment of malignant or benign tumors^8,9^. Recently, investigators also aimed to use HIFU for the treatment of other diseases, such as secondary hypersplenism. Previous studies have investigated the safety and efficacy of HIFU in the treatment of secondary hypersplenism caused by cirrhosis^7^.

In our study, we found that HIFU was safe and effective in the treatment of secondary hypersplenism in most cirrhotic patients whose liver function was classified as Child–Pugh A or B. Postoperatively, subcutaneous ecchymosis of the waist was found in 7 patients (63.6%). Two patients (18.2%) complained of mild pain in the left upper abdomen. Hematologic analysis revealed that HIFU treatment resulted in an approximately 50% increase in the mean PLT count and a 25% increase in the mean WBC count 4 weeks post-HIFU relative to the baseline WBC count pre-HIFU. This result is in accordance with those of previous studies. Zhu et al^7^ reported that HIFU is safe and effective in the treatment of cirrhotic secondary hypersplenism in patients whose liver function is classified as Child–Pugh A or B. No major complications, such as gastrointestinal perforation, peritonitis, splenic rupture or abscess, occurred peri-HIFU and/or post-HIFU. Minor adverse events, such as abdominal pain and dermal ecchymosis, occurred in some patients. The PLT counts, WBC counts and red blood cell (RBC) counts increased after HIFU treatment and peaked 1 year after HIFU. Another study compared the safety and efficacy of HIFU with those of surgical splenectomy and partial splenic embolization (PSE). HIFU was safer and less invasive than splenectomy and traditional PSE^10^. Thus, HIFU seems to be promising in the treatment of cirrhotic secondary hypersplenism. However, few studies have investigated the potential contraindications for HIFU in the treatment of secondary hypersplenism.

The main finding of this study is that multiple small gallstones might be a contraindication for HIFU in the treatment of secondary hypersplenism. However, we enrolled only 11 patients with decompensated cirrhosis. Two of these 11 patients (2/11, 18.2%) with multiple small gallstones developed gallstones that incarcerated in the common bile duct post-HIFU. An earlier related study on patients with cholelithiasis reported that the annual risk of developing an acute biliary complication was 3.1%^11^. Our study found a higher occurrence of acute biliary complications (18.2% vs. 3.1%). The higher occurrence of acute gallstone incarceration into the common duct might be explained by three main reasons. First, we included cirrhotic patients with hypersplenism and gallstones but not patients with gallstones only. It has been proven that changes in bile acid composition and increased nucleation of bile combined with decreased motility of the gallbladder may contribute to the development of gallstones in patients with liver cirrhosis. Thus, cirrhotic patients have a higher incidence and prevalence of gallstones^12–15^. The portal vein diameter and hypersplenism are risk factors for developing gallstones in patients with cirrhosis^15^. Gallstones are more than two times more common in cirrhotic patients with portal hypertension than in cirrhotic patients without portal hypertension^16^. Second, smaller stones, especially stones ≤ 0.55 cm, have been reported to be an independent risk factor for the development of common bile duct stones^17^. This may explain why the two patients with multiple small stones developed common bile duct stones post-HIFU, while the other three patients with a single large gallstone did not experience any related complications. Third, HIFU treatment of the spleen led to coagulation necrosis of the targeted tissues. This phenomenon can directly damage spleen tissues and vessels, which may lead to damage to multiple RBCs in a short time. The latter may result in the production of a large amount of UCB. In addition, perioperative fasting may lead to bile stasis^12^. The CCK level has been reported to be higher in cirrhotic patients than in noncirrhotic patients, which indicates that the gallbladder may be stimulated to contract once a patient starts to eat, especially with a greasy diet. We speculate that the increased level of CCK combined with the large burden of UCB and bile stasis may contribute to the development of common bile duct stones^18,19^.

This study had some limitations. First, the number of patients enrolled was limited. Only 11 patients were included in this study. Decompensated cirrhosis with gallstones incarcerated in the common bile duct indicates that some invasive procedures, such as ERCP, might be required for treatment. However, procedural risks and complications, such as acute-on-chronic-liver failure, have been reported to be quite common in patients with decompensated cirrhosis undergoing ERCP^20^. This suggests that prevention could be much better than salvation. Since two patients with multiple small gallstones developed small stones incarcerated in the common bile duct post-HIFU, before we go further, it is necessary to consider whether we chose the wrong patients for HIFU treatment. Second, this study was not placebo controlled. This was a retrospective study, and the small number of enrolled patients did not allow us to study some patients as controls. However, all 11 enrolled patients were well defined and homogenous. Third, the percentage of ablation required for better outcomes is not clearly stated. In our study, the ablated volume to spleen volume rate was 35%-61%. Up to now, no study has reported the best percentage of HIFU ablation in the treatment of secondary hypersplenism. Thus, this is from the experience of PSE in the treatment of secondary hypersplenism^21^. It has been reported that it could be dangerous when splenic necrosis is over 70% after PSE, as subsequent splenic abscess may lead to death. Finally, the targeted ablated region of the spleen hasn’t been determined yet. In our study, we prefer to choose the middle and/or lower pole of the spleen or the hilum of the spleen in order to avoid stimulating the pleura and/or damaging the adjacent organs. Actually, the small sample size neither allow us to determine the most appropriate ablation spleen volume nor the best targeted ablation area. A large-sample multicenter clinical trial with a long-term follow-up is required to illustrate the proper indications, contraindications, ablation volume as well as the most appropriate ablation area for HIFU in the treatment of secondary hypersplenism.

## Methods

This study was approved by the Institutional Review Board of the Suining Central Hospital in Suining and conducted in compliance with the ethical principles for medical research involving human subjects stated in the World Medical Association Declaration of Helsinki (version 2013). Informed consent was obtained from all subjects. An informed consent form was signed by the patients before HIFU treatment. From October 2019 to May 2021, a total of 11 patients with decompensated cirrhosis accompanied by hypersplenism (8 males and 3 females; average age 54.0 ± 8.6 years) who underwent HIFU ablation of the spleen at the High Intensity Focused Ultrasound Center of Suining Central Hospital were enrolled in this study. They presented with an enlarged spleen on ultrasound or a CT/MRI scan. The diagnosis of hypersplenism was based on an imaging examination and peripheral blood and bone marrow tests. Hypersplenism was defined as splenomegaly, leucopenia (white blood cell [WBC] count < 3 × 10^9^/L), and thrombocytopenia (platelet [PLT] count < 50 × 10^9^/L) (Table 1)^22^. The clinical symptoms and indicators of all patients who underwent HIFU were evaluated before HIFU and closely followed up for 24 h, 2 weeks and 4 weeks after HIFU. Blood counts and liver function tests, abdominal ultrasound and/or CT/MRI scans were performed before HIFU and 24 h, 2 weeks and 4 weeks after HIFU.

### HIFU ablation

HIFU treatment was performed with a JC focused ultrasound tumor therapeutic system (Chongqing Haifu Medical Technology Co., Ltd., Chongqing, China) under real-time sonographic guidance. The device consisted of an ultrasound therapy transducer with an ultrasound generator, a real-time diagnostic ultrasound device, a 6-dimensional movement system, computer units for automated control, a treatment bed, and a degassed water circulation unit. An ultrasound imaging device with a 2.5–3.5 MHz probe was used for real-time imaging guidance.

HIFU treatment was performed under general anesthesia to ensure immobilization during the procedure as well as to prevent superficial skin pain. The patient was positioned prone so that the skin overlying the spleen to be treated could be easily placed in contact with the degassed water. The location, size, and shape of the spleen and adjacent organs were clearly identified on ultrasound. The middle and/or lower pole of the spleen or the hilum of the spleen was usually selected for treatment. The purpose of selecting the middle or lower pole of the spleen or the hilum of the spleen as the targeted ablative area was to avoid stimulating the pleura and/or damaging the adjacent organs. Regarding the specific ablative scheme used, the linear scanning mode for HIFU exposure was prescribed. This ablated the targeted tissue from a point to a plane and from deep to the surface. The grayscale change was obtained from the diagnostic images to identify and monitor the extent of treatment (Fig. 1). The vital signs of the patient were observed closely during the whole procedure. The treatment power was gradually increased to 300 to 400 W. One sonication lasted for 5 s. The real-time sonograms obtained before and after each exposure were immediately compared to determine whether the hyperechoic region indicating the extent of coagulation necrosis had covered the desired treatment area. The HIFU parameters for hypersplenism were as follows: therapy frequency 0.98 MHz, focal length 160 mm, therapeutic power 300–400 W, and average ablation time 2788 s (range 1100–5039 s).

### Statistical analysis

All data are presented as the mean ± standard deviation. Data were analyzed with SPSS 25.0 software (Statistical Product and Service Solutions, Chicago, IL, USA) using a paired-samples *t* test.

## Data Availability

The raw data obtained and analyzed in this study are available from the corresponding author upon reasonable request.
